# Red Blood Cell Fatty Acids and Incident Diabetes Mellitus in the Women’s Health Initiative Memory Study

**DOI:** 10.1371/journal.pone.0147894

**Published:** 2016-02-16

**Authors:** William S. Harris, Juhua Luo, James V. Pottala, Karen L. Margolis, Mark A. Espeland, Jennifer G. Robinson

**Affiliations:** 1 Department of Internal Medicine, Sanford School of Medicine, University of South Dakota, and OmegaQuant Analytics, LLC, Sioux Falls, South Dakota, United States of America; 2 Department of Epidemiology and Biostatistics, School of Public Health, Indiana University, Bloomington, Indiana, United States of America; 3 Department of Internal Medicine, Sanford School of Medicine, University of South Dakota, Sioux Falls, South Dakota, United States of America; 4 HealthPartners Foundation for Education and Research, Minneapolis, Minnesota, United States of America; 5 Department of Biostatistical Sciences, Wake Forest School of Medicine, Winston-Salem, North Carolina, United States of America; 6 Department of Internal Medicine, College of Medicine, University of Iowa, Iowa City, Iowa, United States of America; German Diabetes Center, Leibniz Center for Diabetes Research at Heinrich Heine University Duesseldorf, GERMANY

## Abstract

**Context:**

The relations between dietary and/or circulating levels of fatty acids and the development of type 2 diabetes is unclear. Protective associations with the marine omega-3 fatty acids and linoleic acid, and with a marker of fatty acid desaturase activity delta-5 desaturase (D5D ratio) have been reported, as have adverse relations with saturated fatty acids and D6D ratio.

**Objective:**

To determine the associations between red blood cell (RBC) fatty acid distributions and incident type 2 diabetes.

**Design:**

Prospective observational cohort study nested in the Women’s Health Initiative Memory Study.

**Setting:**

General population.

**Subjects:**

Postmenopausal women.

**Main Outcome Measures:**

Self-reported incident type 2 diabetes.

**Results:**

There were 703 new cases of type 2 diabetes over 11 years of follow up among 6379 postmenopausal women. In the fully adjusted models, baseline RBC D5D ratio was inversely associated with incident type 2 diabetes [Hazard Ratio (HR) 0.88, 95% confidence interval (CI) 0.81–0.95) per 1 SD increase. Similarly, baseline RBC D6D ratio and palmitic acid were directly associated with incident type 2 diabetes (HR 1.14, 95% CI 1.04–1.25; and HR 1.24, 95% CI 1.14–1.35, respectively). None of these relations were materially altered by excluding incident cases in the first two years of follow-up. There were no significant relations with eicosapentaenoic, docosahexaenoic or linoleic acids.

**Conclusions:**

Whether altered fatty acid desaturase activities or palmitic acid levels are causally related to the development of type 2 diabetes cannot be determined from this study, but our findings suggest that proportions of certain fatty acids in RBC membranes are associated with risk for type 2 diabetes.

## Introduction

The role that dietary and/or circulating fatty acid levels (typically expressed as a percent of total fatty acids) may play in the development of type 2 diabetes mellitus (T2DM) is unclear. Dietary intake of marine omega-3 (n-3) fatty acids (eicosapentaenoic acid, EPA and docosahexaenoic acid, DHA) based on food frequency questionnaires was inversely associated with incident T2DM in a study from China [1], but directly associated with incident disease 3in the Women’s Health Study [2]. A 2012 meta-analysis of prospective cohort studies concluded that there was a non-significant 10–15% increased incidence of T2DM when comparing the highest to the lowest levels of intake of fish or omega-3 fatty acids [3]. However, biomarker-based studies have shown either no or an *inverse* association between omega-3 levels and incident T2DM (Table 1). These studies have used plasma, plasma phospholipids and/or red blood cell (RBC) fatty acid distributions as markers of exposure. In these studies, a variety of fatty acids were inversely associated with risk for T2DM, including short- and long-chain omega-3 fatty acids, *trans*-palmitoleic and odd chain saturated fatty acids (markers of dairy consumption), linoleic acid (LA), 8oleic and eicosadienoic acids among others. Similarly, risk was directly associated with fatty acids such as stearic, palmitic, palmitoleic and dihomo-γ-linolenic (DGLA) acids.

**Table 1:** Review of literature examining associations between fatty acid biomarkers and incident type 2 diabetes mellitus.

Author Year	N^a^	Mean Age (% Males)	Years Follow-up	Biomarker	Covariates	Fatty Acid Associations (inverse) direct^b^
Vessby[4] 1994	1828 (4.1%)	50 (100%)	10.2	Plasma CE	BMI	**(18:1n9)**
						20:3n6, 16:1n7, 18:0
Wang[5] 2003	2909 (8.7%)	54 (49%)	9	Plasma CE,	Age, sex, BMI, waist-hip ratio, F/H T2DM, smoking, education, alcohol	**(18:2n6)**
						16:0, 16:1n7, 20:3n6, 20:4n6
				Plasma PL		**(18:2n6, 18:1n9, 18:3n3)**
						16:0, 18:0
Hodge[6] 2007	3737 (9.7%)	55 (44%)	4	Plasma PL	Age, sex, birth country, F/H T2DM, BMI, physical activity, alcohol, waist-hip ratio	**(15:0, “trans”, 18:2n6, D5D)**
						18:0, 16:1n7, 20:3n6, D6D
Krachler[7] 2008	159/291	52 (?)	5.4	RBC	Age, sex, alcohol intake	**(15:0, 17:0, 18:2n6, 22:5n3, D5D)**
						16:0, 18:0, 16:1n7, 20:3n6, D6D
					+BMI, hemoglobin A1	**(15:0, 17:0)**
						22:4n6
Mozaffarian[8] 2010	3736 (8%)	75 (45%)	14	Plasma PL	Age, sex, race, site, BMI, waist, physical activity, education, smoking, alcohol, prevalent CHD and intake of carbohydrates, protein, red meat, dairy, kcalories	**(16:1n7 trans)** only FA examined
Mozaffarian[9] 2010	3630 (8.2%)	75 (45%)	9.3	Plasma PL	Age, sex, race, BMI, leisure time activity, education, smoking, alcohol, prevalent CHD, prevalent T2DM, and intake of carbohydrates and protein	Only FA examined was 16:1n7; no relations with incident T2DM were found
Patel[10] 2010	199/184	64 (47%)	10	RBC	Age, sex, F/H T2DM, BMI, smoking, physical activity, alcohol	**(20:2n6, D5D)**
						16:0, 16:1n7, D6D
				Plasma PL		**(18:0, 18:1n7, 20:1n9, 18:2n6, 20:2n6, D5D)**
						16:0, D6D
Djousse[11] 2011	3088 (6.6%)	75 (39%)	10.6	Plasma PL	Age, sex, race, BMI, physical activity, alcohol, smoking, LDL-cholesterol, plasma PL 18:2n6	**(20:5n3+22:6n3, 18:3n3)** only FA examined
Kroger[12] 2011	2724 (25%)	51 (43%)	7	RBC	Age, sex, BMI, waist, bicycling, sports activity, education, smoking, alcohol, work activity, coffee, fiber	**(20:0, 18:2, D5D)**
						24:0, 16:1n7, 16:1n9, 18:3n6, 20:3n6, D6D
Zong[13] 2013	2066 (24%)^c^	58 (46%)	6	RBC	Age, sex, region, residence, physical activity, educational attainment, current smoking, current drinking, F/H of diabetes, total energy intake, % of energy intake from carbohydrate, and energy-adjusted dietary glycemic index	16:0, 16:1n7
Mahendren[14] 2014	735 (4%)	55 (100%)	5	RBC	Age, sex, physical activity	**(18:2n6)**
						16:1n7
Virtanen[15] 2014	2212 (19%)	53 (100%)	19.3	Serum	Age, exam year, BMI, F/H type 2 diabetes, smoking, education, leisure time activity, alcohol, serum 18:2n6	**(20:5n3+22:5n3+22:6n3, 22:5n3, 22:6n3)**
Forouhi[16] 2014	12,403/ 16,154	52 (38%)	11.7	Plasma PL	Age, exam year, center, sex, BMI, education, smoking, physical activity, alcohol	**(15:0, 17:0, 23:0, 20:0, 22:0, 24:0)**
						14:0, 16:0, 18:0
Ma[17] 2015	3004 (10%)	74 (40%)	10.2	Plasma PL	Age, sex, race, education, clinic, smoking status, alcohol consumption, leisure time physical activity, prevalence of ischemic heart disease, hypertension at baseline, BMI, waist circumference, and consumption of carbohydrate and of protein (percentages of energy), and total energy.	**(18:1n7)**
						16:0, 18:0
Wang[18] 2015	2919 (9.8%)	74 (39%)	18	Plasma PL	Age, sex, race, education, enrollment site, BMI, waist circumference, smoking status, alcohol intake, physical activity, CVD, and hypertension; secondary adjustment for plasma PL 16:0 and 18:0	**(16:1n7 trans)** None directly associated with base adjustment; 16:1n9 trans and 18:1 trans with secondary adjustment
Lankinen[19] 2015	1302 (5%)	55 (100%)	5.9	Plasma PL (CE, TG)	Age, BMI, smoking, physical activity and fasting glucose	D6D, 20:3n6

^a^ Cohort size (% events) or cases/controls;

^b^ Statistically significant associations as defined by each author; fatty acid names given in Table 4.

^c^ personal communication. Abbreviations: F/H, family history; RBC, red blood cell; PL, phospholipids; BMI, body mass index; CHD, coronary heart disease; D5D, delta-5 desaturase ratio; D6D, delta-6 desaturase ratio, T2DM, type 2 diabetes mellitus

Other fatty acid-based metrics that have been linked with the development of T2DM are “desaturase ratios.” [6, 7, 10, 20] The delta-5 and delta-6 desaturases (D5D and D6D, respectively) are hepatic enzymes that, in concert with a suite of elongases, add new double bonds (and 2-carbon units) to the growing fatty acid chain. They thus control the conversion of shorter-chain essential fatty acids (LA and alpha-linolenic) to their respective longer-chain metabolites; DGLA and arachidonic acid (AA) from the former, and EPA, DHA and n-3 docosapentaenoic acid from the latter. As the activities of these hepatic enzymes cannot be measured directly, they are conventionally estimated from product/precursor ratios measured in a number of fatty acid pools [e.g., plasma, plasma phospholipids / cholesteryl esters, red blood cells (RBCs)]. D5D activity is estimated from the ratio of AA to DGLA, and D6D is estimated from the ratio DGLA to LA. In the 5 studies (Table 1) that have explored the connection between the desaturase ratios and risk for incident T2DM [6, 7, 10, 12, 14], all but one [14] reported that D5D was inversely and/or D6D directly related to risk. Another metric derived from structural equation modeling of RBC fatty acid profiles, the “PUFA (polyunsaturated fatty acid) factor,” that has been suggested as a single metric accounting for the inter-correlated nature of overall essential fatty acid patterns [21] was also examined for its relations with incident T2DM. It includes three omega-3 (alpha-linolenic, EPA and DHA) and three omega-6 (AA, adrenic, and n-6 docosapentaenoic acids) PUFAs.

We recently reported the RBC fatty acid composition in the Women’s Health Initiative Memory Study (WHIMS), a large cohort of postmenopausal women in the United States[22]. With about 11 years of follow-up in approximately 6400 women, this study afforded us the opportunity to explore the relations between RBC fatty acids and incident T2DM. Our primary hypotheses were that the risk for incident disease would be inversely associated with RBC D5D, omega-3 index (RBC EPA+DHA [23]), LA, and the PUFA factor; and directly associated with D6D. In an exploratory analysis, we also examined the relations of all RBC fatty acids with incident disease.

## Methods

### Subjects

All subjects were participants in the WHIMS randomized trials which examined the effects of postmenopausal hormone therapy on cognitive function in women aged 65–80 years. [24, 25] Recruitment began in 1995. All provided signed informed consent, and the study was approved by the National Institutes of Health and the institutional review boards of the 50 participating centers. Of the 7479 women enrolled in the WHIMS trials, baseline RBC fatty acid measurements were available from 7299 (98%). The following were excluded: 486 had diabetes at baseline, 16 were lost to follow-up, 192 had RBC fatty acid data that was technically unusable [22], and 226 were missing covariates. Thus, 6379 women were included in the analysis.

### RBC Fatty Acid Analyses

RBC membrane fatty acid composition was analyzed using gas chromatography with flame ionization detection, and expressed as a weight percent of total identified fatty acids [22]. The inter-assay coefficient of variation for the primary fatty acids of interest was <6.5%. During the aliquoting phase, the RBC samples were stored improperly at -20°C for a period of approximately two weeks, causing oxidative degradation of the PUFAs before measurement. The original fatty acid levels were estimated with multiple imputations using independent data on fatty acid degradation rates and the length of time the samples were exposed to -20°C. 21[22]

### Assessment of Diabetes Status

Incident T2DM was defined as a positive answer to the question (asked annually) regarding “newly prescribed treatment for diabetes with pills or insulin shots”. The date of diabetes onset was assigned as the midpoint between the dates between the survey when diabetes was self-reported and the previous survey. Follow-up was right-censored at the study close-out date of August 2009. Self-reported diabetes in the WHI has been found to be a reliable indicator of diagnosed diabetes with a positive predictive value of 82.2% based on medication inventories, fasting glucose levels and medical record review. [26, 27] Few women who did not self-report diabetes were found to have diabetes on medical record review (negative predictive value 94.5%). False negative self-reports included women who were undiagnosed, unaware of their diagnosis or treated with lifestyle alone.

### Statistical Analyses

Chi-squared tests were used to evaluate univariate differences for categorical variables between patients that developed diabetes and those who did not, and t-tests were used for continuous variables. Cox proportional hazards (PH) regression models were used to estimate hazard ratios (HR) and 95% confidence intervals (CI) for the associations between the RBC fatty acid biomarkers (log-transformed value) and the risk of T2DM. Our primarily hypotheses included 5 fatty acid metrics: the omega-3 index, LA, D5D, D6D and the PUFA factor. For each fatty acid metric, first, we constructed an age- and race- adjusted model (using categories of Asian or Pacific Islander, Black or African-American, Hispanic/Latino, non-Hispanic white, or other). We then fitted a fully adjusted model with the addition of education (high school or less, some college/technical training, college or some post-college, master or higher), current smoking status (Yes/No), recreational physical activity (total metabolic equivalent tasks [METs] per week), alcohol intake >7 drinks/wk, Yes/No), waist girth (cm), dietary glycemic load, and family history of diabetes (Yes/No). Potential hormone therapy treatment effects due to randomized assignment (0.625 mg conjugated equine estrogen or matching placebo; 0.625 conjugated equine estrogen + 2.5 mg medroxyprogesterone acetate or matching placebo) in the two WHIMS trials were included as four strata in the Cox PH regression models[28]. Due to the fatty acid levels being corrected via multiple imputations, we properly accounted for the covariance in the imputed fatty acid values in the analysis [22]. An interaction between age (dichotomized at its mean value), waist circumference, and race with each of the 5 primary fatty acid metrics was added to the fully adjusted model to test if these factors modified the associations with incident diabetes using the Wald test. To limit effects of possible reverse causation, we also performed supporting analyses after excluding the first 2 years of follow-up. Another sensitivity analysis excluded those women with baseline glucose levels > 125 mg/dL (n = 240). The proportional hazards assumption was tested using cumulative sums of Martingale residuals. [29]

Cumulative hazards were also estimated by quintile of the RBC fatty acid metrics based on the Nelson-Aalen method. The log-rank statistic was used to test for homogeneity among the quintiles which were then included in the minimally-adjusted and the fully-adjusted models to estimate respective hazard ratios. All tests were 2-sided, and a critical level alpha = 0.01 was used for testing the five hypothesized FA metrics. A critical level ≤0.0022 (0.05 / 23 fatty acids) was used for exploratory analysis involving all other individual fatty acids based on Bonferroni correction method to adjust for multiple testing. All statistical analyses were conducted by using SAS, version 9.3, software (SAS Institute, Inc., Cary, North Carolina).

## Results

The median follow-up was 11 years (interquartile range, 7.8–11.9), and in that time, there were 703 cases of incident T2DM. Those participants who developed T2DM were more likely to be of African American, to be overweight, to have a larger waist circumference, to exercise less, to drink alcohol less frequently and to have a family history of the disease (Table 2). Of the five primary RBC fatty acid metrics of interest, unadjusted differences were observed only for the D5D and D6D ratios; D5D was lower and D6D was higher in those who developed T2DM during follow-up.

**Table 2:** Participant’s Characteristics by Incident Diabetes during 11-years Median Follow-Up [Interquartile Range (7.8–11.9 years)].

	Incident Diabetes		
	No (N = 5676)	Yes (N = 703)	P value^1^
Age at baseline [years]: mean (SD)	70.2 (3.9)	69.8 (3.7)	0.02
Race: n (%)			0.0007
Asian or Pacific Islander	89 (1.6)	14 (2.0)	
Black or African American	333 (5.9)	69 (9.8)	
Hispanic or Latino	124 (2.2)	91 (2.7)	
Non-Hispanic White	5036 (89)	587 (84)	
Other	94 (1.7)	14 (2.0)	
Highest Education: n (%)			0.51
High School or Less	1667 (29)	218 (31)	
Some College or Technical School	2273 (40)	276 (39)	
Bachelor’s Degree	1002 (18)	130 (18)	
Master’s Degree or Higher	734 (13)	79 (11)	
Body Mass Index [kg/m^2^]: n (%)			<0.0001
< 25	1842 (32)	109 (16)	
[25–30)	2136 (38)	246 (35)	
[30–35)	1126 (20)	213 (30)	
[35–40)	415 (7.3)	86 (12)	
≥ 40	157 (2.8)	49 (7.0)	
Waist circumference [cm]: mean (SD)	87 (13)	94 (14)	<0.0001
Physical activity [METs/week]: mean (SD)	12 (14)	10 (13)	0.01
Dietary glycemic load^2^: mean (SD)	101 (44)	103 (47)	0.22
Alcohol intake >7 drinks/week: n (%)	756 (13.3)	45 (6.4)	<0.0001
Current smoking: n (%)	399 (7.0)	44 (6.3)	0.45
Family history of diabetes: n (%)	1682 (30)	322 (46)	<0.0001
Primary RBC fatty acids (FA)			
18:2n-6 (Linoleic, LA; % of total FA)	11.97 (1.79)	11.86 (1.77)	0.12
Omega-3 Index^3^ (% of total FA)	5.28 (1.66)	5.19 (1.53)	0.19
D5D (delta-5 desaturase ratio)^4^	10.19 (2.98)	9.71 (2.85)	0.0002
D6D (delta-6 desaturase ratio)^5^	0.15 (0.04)	0.16 (0.04)	<0.0001
PUFA Factor^6^	-0.49 (0.91)	-0.54 (0.86)	0.16

^1^ A Chi-squared test was used to evaluate differences for categorical variables, and a t-test was used for continuous variables. All t-tests used the logarithm transformation for the fatty acid biomarkers, and a critical level alpha = 0.01 was used for testing the five a priori hypothesized FA metrics (LA, Omega-3 Index, D5D, D6D, PUFA).

^2^ Glycemic load was defined as an indicator of blood glucose induced by an individual’s total carbohydrate intake. Each unit of glycemic load represents the equivalent of 1 g carbohydrate from white bread.

^3^calculated as RBC EPA+DHA.

^4^calculated as AA/DGLA.

^5^calculated as DGLA/LA.

^6^The PUFA factor is comprised of weighted proportions of three n-6 and three n-3 fatty acids (19).

**Fig 1:**
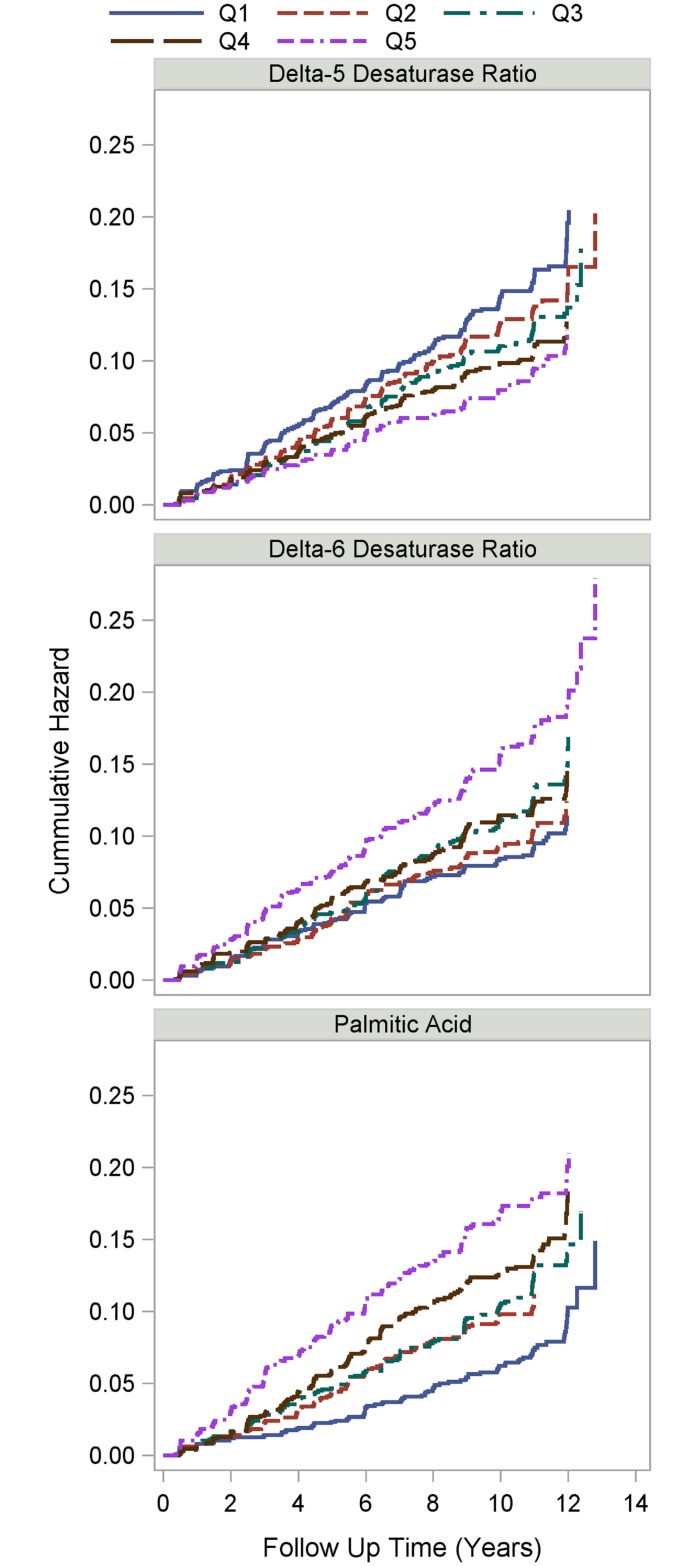
Cumulative Nelson-Aalen hazard estimates of incident diabetes by baseline delta-5 desaturase ratio (top), delta-6 desaturase ratio (middle) and palmitic acid level (bottom). Log Rank p-values = 0.0007, <0.0001 and <0.0001, respectively.

Results from adjusted Cox PH regression models are shown in Table 3 for the D5D and D6D ratios. In all models, 1 standard deviation (SD) higher levels in the D5D ratio were associated with risk reductions that ranged from a 11–18% (minimum p = 0.009). Similarly, in all models using the D6D ratio, the relative risk for incident T2DM over 11 years of follow-up was increased by 13–24% per 1 SD (minimum p = 0.02). Cumulative hazard estimates by quintile of each desaturase ratio supported the continuous variables’ model estimates. The estimated incident rates for T2DM at 10 years of follow-up for D5D were (for quintiles 1 thru 5) 14.4%, 12.6%, 11.0%, 9.9%, and 8.0%, respectively (Log Rank p = 0.0007); and for D6D they were 8.4%, 9.4%, 11.1%, 11.5%, and 15.6% (Log Rank p <0.0001) (Fig 1).

**Table 3:** Multivariable-adjusted association of RBC fatty acid desaturase ratios on the risk of incident diabetes mellitus over 11-years median follow up (per 1 SD increase in desaturase ratio). All models included 4 strata for hormone therapy trial randomization arm. The fully adjusted model included: age, race, waist circumference, highest education, current smoking status, physical activity, weekly alcohol intake, glycemic load, and family history of diabetes with categories listed in Table 2. All 10 imputed fatty acid values were used for multiple imputation inference.

N = 6,379 with 703 events	Hazard Ratio	95% Confidence Limits		P-value
**Log(Delta-5 Desaturase ratio)**				
Model 1: Age, Race adjusted	0.82	0.76	0.89	<0.0001
Model 2: Fully adjusted	0.88	0.81	0.95	0.0017
Model 3: Model 2 with first 2 years of follow up excluded (i.e. 107 events, 79 censored)	0.89	0.81	0.97	0.0086
**Log(Delta-6 Desaturase ratio)**				
Model 1: Age, Race adjusted	1.24	1.14	1.36	<0.0001
Model 2: Fully adjusted	1.14	1.04	1.25	0.0058
Model 3: Model 2 with first 2 years of follow up excluded (i.e. 107 events, 79 censored)	1.13	1.02	1.24	0.018

The numbers of subjects at risk during follow-up are shown in S1 Table. In sensitivity analysis excluding women with baseline hyperglycemia, the HR for D5D was 0.86 (95% CI 0.78, 0.95; p = 0.002), and for D6D 1.17 (95% CI 1.05, 1.31; p = 0.004) in the fully adjusted models. In secondary analysis, there were no interactions with age, waist circumference, or race with any of the primary fatty acid metrics for incident T2DM. (S2 Table)

Additional exploratory analyses were undertaken with all of the individual RBC fatty acids using a Bonferroni adjusted critical level of ≤ 0.0022; hazard ratios are shown in Table 4 per a 1 SD increase in logarithm transformed fatty acid value. In fully adjusted models, there were two significant findings, palmitic acid (C16:0) and palmiotoleic acid (C16:1) were directly associated with increased risk of T2DM. The most notable HR was with palmitic acid which had a 24% increased relative risk to develop T2DM over 11 years follow-up per 1 SD higher levels in fully adjusted models. In the sensitivity analysis with only normoglycemic women, the HR was 21% increased (p = 0.003). Cumulative hazard estimates of palmitic acid at 10 years of follow-up increased by quintile (6.2%, 9.8%, 10.5%, 12.7%, and 16.9%, for quintiles 1 thru 5, respectively; Log Rank p < 0.0001, Fig 1). Hence, the RBC fatty acid (or metric) most strongly related to incident T2DM in this study was palmitic acid. Compared with a palmitic acid value of < 19.0% (lowest risk; 1st quintile), patients were almost 2 times as likely to develop T2DM over the 11 years of follow-up (HR = 1.83; 95% CI: 1.39–2.40, p<0.0001) with RBC palmitic acid values > 22.4% (highest risk, 5^th^ quintile; p-value for trend <0.001; Table 5). In a sensitivity analysis excluding women with elevated glucose in the fully adjusted model, the HRs were reduced by 5–10% at each quintile, leaving only the highest quintile of palmitic different from the lowest (HR 1.66, p<0.002), and reducing the p-value for trend from <0.0001 to 0.001. For palmitoleic, the fully adjusted HR of 1.15 (p = 0.0019; Table 4) was unchanged (1.16) in the sensitivity analysis, but the p-value was slightly attenuated (p = 0.0027).

**Table 4:** Hazard ratios (95% CI) for incident type 2 diabetes over 11 years median follow-up in fully-adjusted models using log fatty acid values (per 1 SD). A significance level alpha ≤ 0.0022 (0.05/23) was used based on Bonferroni correction method to adjust for multiple testing. All models included 4 strata for hormone therapy trial randomization arm. The fully adjusted model included: age, race, waist circumference, highest education, current smoking status, physical activity, weekly alcohol intake, glycemic load, and family history of diabetes with categories listed in Table 2. All 10 imputed fatty acid values were used for multiple imputation inference.

	HR	95% CI		P values
14:0 (Myristic)	1.13	1.04	1.24	0.0070
16:0 (Palmitic)	1.24	1.14	1.35	**<0.0001**
18:0 (Stearic)	0.95	0.85	1.06	0.34
20:0 (Arachidic)	0.92	0.83	1.01	0.088
22:0 (Docosanoic)	0.96	0.89	1.05	0.39
24:0 (Lignoceric)	0.98	0.89	1.07	0.60
16:1 (Palmitoleic)	1.15	1.05	1.26	**0.0019**
18:1 (Oleic)	0.96	0.88	1.05	0.35
20:1 (Eicosenoic)	0.96	0.88	1.04	0.28
24:1 (Nervonic)	1.04	0.87	1.06	0.41
16:1t (trans Palmitoleic)	0.95	0.87	1.04	0.24
18:1t (trans Oleic)	0.90	0.83	0.98	0.021
18:2t (trans Linoleic)	0.99	0.90	1.08	0.77
18:3n-6 (γ-Linolenic)	1.08	0.99	1.18	0.093
20:2n-6 (Eicosadienoic)	0.91	0.82	1.02	0.11
20:3n-6 (Dihomo-γ-linolenic, DGLA)	1.13	1.04	1.24	0.0057
20:4n-6 (Arachidonic, AA)	0.94	0.85	1.03	0.16
22:4n-6 (Adrenic)	0.99	0.90	1.08	0.78
22:5n-6 (Docosapentaenoic n-6)	1.04	0.95	1.15	0.38
18:3n-3 (α-Linolenic)	1.00	0.91	1.10	0.96
20:5n-3 (Eicosapentaenoic, EPA)	0.95	0.87	1.03	0.23
22:5n-3 (Docosapentaenoic n-3)	0.90	0.82	0.99	0.028
22:6n-3 (Docosahexaenoic, DHA)	0.99	0.90	1.08	0.77

**Table 5:** Association of RBC fatty acid palmitic acid (C16:0) quintiles on the risk of incident diabetes mellitus over 11-years median follow up.

Palmitic Acid [% of total fatty acids]	Hazard Ratio	95% Hazard Ratio Confidence Limits		P-value*	Incident Rate per 100 person-year
**Model stratified for hormone therapy trial randomization arm only**					
≤ 19.0%	1.00	-	-	-	0.82
(19.0% to 20.2%]	1.22	0.83	1.80	0.29	1.00
(20.2% to 21.2%]	1.41	1.01	1.95	0.041	1.15
(21.2% to 22.4%]	1.51	1.03	2.24	0.038	1.24
> 22.4%	1.85	1.40	2.43	<0.0001	1.50
**Fully adjusted model**					
≤ 19.0%	1.00	-	-	-	Same as above
(19.0% to 20.2%]	1.21	0.81	1.80	0.34	
(20.2% to 21.2%]	1.38	1.00	1.90	0.052	
(21.2% to 22.4%]	1.49	1.02	2.18	0.040	
> 22.4%	1.83	1.39	2.40	<0.0001	

* p-value for trend <0.001. All models included 4 strata for hormone therapy trial randomization arm. The fully adjusted model included: age, race, waist circumference, highest education, current smoking status, physical activity, weekly alcohol intake, glycemic load, and family history of diabetes with categories listed in Table 2. All 10 imputed fatty acid values were used for multiple imputation inference. Note that the (x to y] designation indicates that x is included in the indicated range but that y is not; it is the x value in the next range.

## Discussion

The purpose of this study was to determine the extent to which a variety of RBC-based fatty acid metrics were associated with incident T2DM over 11 years of follow-up. We originally examined five metrics, two of which, D5D and D6D, we found to be significantly related to risk for diabetes, the former inversely and the latter directly. The other three metrics (the omega-3 index, the PUFA factor, and LA) were not related in fully-adjusted models, with the exception that the omega-3 index was inversely associated with risk in women under 70 years of age. Further analysis of all individual RBC fatty acids revealed that palmitic and palmitoleic acids were directly related to risk for incident disease, the strongest of which was palmitic acid (see below).

Connections between fatty acids and diabetes have been explored in many settings with several different study designs (see review by Riserus et al. [30]). Since dietary intake surveys provide only a rough estimate of *in vivo* fatty acid status (stronger relations for some and weaker for other fatty acids [31]), the use of circulating fatty acid levels as biomarkers of status is preferred. [32] In the study of incident T2DM, biomarker-based studies have more commonly found significant relations with disease than diet survey-based approaches when compared in the same contexts. [6, 10–12, 17, 33]

Fourteen previous reports have been published on the relations between fatty acid biomarkers and incident T2DM (Table 1). Most took a discovery approach and examined a relatively full suite of fatty acids, whereas others use a hypothesis-based approach and focused on a few specific fatty acids. As is evident from the Table, the present study is the second largest to date (but far smaller than the EPIC-InterACT study [16]) and is the only one done nested in a randomized controlled trial for hormone therapy in postmenopausal women. The fatty acids or metrics most consistently reported to be adversely associated with incident diabetes across these studies are palmitoleic acid, palmitic acid, and the D6D ratio; and to be favorably associated, LA and the D5D ratio. These, along with the omega-3 fatty acids, will be discussed below.

### Desaturase ratios and related fatty acids (AA/DGLA and DGLA/LA)

Among the most consistent fatty acid metrics associated with risk for T2DM are the desaturase ratios. Of the six past studies that examined them, five found significant inverse relations with disease for the D5D ratio and/or direct relations for the D6D ratio [6, 7, 10, 12, 19]; and the study that did not find these associations included only 30 events [14]. Consistent with these findings, Warensjo, et al. [34] found direct relations for D6D and inverse relations for D5D and the development of metabolic syndrome over 20 years. The extent to which these ratios actually reflect hepatic enzyme activities is unclear, nevertheless, as circulating fatty acid-based biomarkers, their relations with future T2DM appear to be robust. In intervention studies with fish oil, D6D was reduced and D5D was increased [35], but these ratios were unchanged by differences in total dietary fat [36]. The RBC D5D and D6D ratios are highly correlated (r = 0.68) which is not unexpected since DGLA is in the denominator in the former and the numerator of the latter. Indeed, DGLA alone [6, 7, 12] and LA alone [6, 7, 10, 12, 14, 37] were commonly associated with risk, and here we observed an association of DGLA with incident disease (p<0.01, although this did not meet the <0.002 criterion for multiple testing). AA alone was never found to be associated with risk for T2DM. What these findings imply is that factors that lower DGLA levels—either by enhancing conversion to AA or by slowing conversion from LA, may favorably influence metabolic pathways involved with the development of diabetes. Whether DGLA metabolites, such as the 1 series prostaglandins or other oxylipins [38], may have adverse effects (e.g., higher DGLA is associated with higher CRP and lower adiponectin levels [39]), or AA and/or LA metabolites favorable effects remains to be seen., Diets rich in LA (e.g., 14% en) lower levels of DGLA and AA in serum CE, whereas diets very low in LA (e.g., <2% en) increase levels of the longer-chain metabolites [40]. These fatty acid distributions may simply be epiphenomena caused by dysglycemic processes that incidentally up-regulate the *FADS2* gene and at the same time down-regulate the *FADS1* gene (which code for D6D and D5D, respectively). A recent exploration of the potential T2DM factors associated with the desaturase ratios in the EPIC study suggested that liver fat accumulation, but not high density lipoprotein cholesterol, adiponectin or C-reactive protein, may be mediating the relationship [41]. Regardless of the question of causality, the RBC D5D and D6D ratios have the potential to risk-stratify patients for T2DM.

### Palmitic acid

RBC palmitic acid gave the strongest signal with incident T2DM of all the fatty acids examined here, confirming the findings of others [7, 10, 13, 16]. Although the third most prevalent fatty acid in the diet (about 20% of total), its levels in RBCs do not correlate well with intake [10, 12, 42] nor do they respond proportionally to changes in intake [42, 43].8 This is largely because palmitic acid is also an “endogenous” fatty acid synthesized *de novo* from the products of carbohydrate metabolism. Nevertheless, considerable evidence has accumulated that diets rich in saturated fatty acids (about 2/3^rds^ of which in the US come from palmitate) and carbohydrates and low in unsaturated fatty acids increase insulin resistance, perhaps via their up-regulation of lipogenic and suppression of fatty acid oxidative pathways (see review by Riserus [30]) which can lead to hepatic steatosis [44] and to metabolic syndrome [13]. Palmitate has also been shown to stimulate ceramide synthesis in skeletal muscle which increases tissue insulin resistance [45, 46].

### Palmitoleic Acid

In contrast to *trans* palmitoleic (produced by ruminant bacteria and derived largely from dairy products [47]) which was inversely related to risk for incident T2DM in the Cardiovascular Health Study, [8] *cis* palmitoleic was directly associated with incident T2DM in our study, which derives mostly from hepatic and adipose synthesis. [48] It is increased with high-carbohydrate diets [36, 42] and a marker of de novo lipogenesis [49]. Palmitoleic was directly related to risk in 8 of the 9 previous studies in which it was examined. The present study confirms these findings. It had been hypothesized [9] that, based on considerations about the potential feedback inhibition of adipose-derived palmitoleic acid on hepatic lipogenesis, higher levels would be associated with improved glycemic status and reduced risk for T2DM, but as noted, this has not proven to be the case. Diets with a high ratio of polyunsaturated to saturated fatty acids (largely LA to palmitic) alters cell membrane biophysics [50], and improve binding of insulin to skeletal muscle nuclei and stimulate glucose transport [51].

### Omega-3 fatty acids

As noted, overall the omega-3 index was not associated with incident T2DM, but in women under 70 (mean age of the cohort) it may be beneficial. Djousse et al. [11] and Virtanen et al. [15] are the only others to report an association between incident T2DM and long-chain omega-3 fatty acids (measured in plasma phospholipids or serum, respectively). The Cardiovascular Health Study cohort [11] was of similar age to ours and follow up of similar duration but contained 42% males; how this may have affected these relations is not clear since sex was adjusted for in their model. EPA+DHA was not significantly associated with incident disease until low density lipoprotein cholesterol and plasma phospholipid LA levels were added to the model, and family history of T2DM and education were not included. We also confirmed an earlier observation [15] that docosapentaenoic acid (n-3), the metabolic intermediate between EPA and DHA, is beneficially associated with incident disease. Although this fatty acid may be storage form of EPA (by retroconversion), little is known about the physiological effects of this fatty acid, much less how it might be involved with diabetes 34. The inconsistency among biomarker-based studies regarding relations between omega-3 and T2DM is somewhat reflected in the heterogeneity seen among diet-based studies [52] where an increase of one serving of fish per week was associated with a 5% increased risk for T2DM in six US cohort studies, with no change in three, and with a 2% reduction in risk in five studies from Asia/Australia. Clearly, the relations between omega-3 fatty acid biomarkers and T2DM remains unclear.

### Other fatty acids

No other fatty acids (besides palmitic and palmitoleic) were significantly associated with incident T2DM after adjusting for multiple testing. Two fatty acids with p-values <0.01 merit comment, however. DGLA (mentioned above) and myristic acid. The latter was directly associated with incident diabetes supporting the findings from a large European study [16]. Myristic is an endogenous fatty acid reflecting, along with palmitic and palmitoleic acids, *de novo* lipogenesis [53] with some arising from palmitate oxidation and/or laurate elongation [54]. In another study RBC myristic levels were marginally directly related to the risk of developing metabolic syndrome, they were not associated with incident T2DM [13].

### Cross sectional relations of fatty acids with pre-diabetes markers

Differences in desaturase ratios in dysglycemic patients reported in cross sectional studies [39, 55–57, 40, 41] suggest that the disease process itself may be altering fatty acid metabolic pathways. Thus, a reverse causation situation could be occurring whereby patients with elevated D6D and/or depressed D5D ratios at baseline may already have “subclinical” T2DM. The loss of the significant relations between the desaturase ratios and incident T2DM when baseline hemoglobin A1c was included in the models in one study also supports this possibility [7]. In order to at least partially control for this, a sensitivity analysis was conducted in which we eliminated all incident cases of T2DM in the first two years after the RBC samples were collected. This did not alter the significant relations with these two ratios seen in the entire cohort suggesting that reverse causation was not at play here.

Certain limitations should be noted, such as unmeasured confounding. Also, the use of only a single measure of RBC FA content at baseline (instead of serial assessments over the 11 year period) reduced our ability to accurately describe the long-term exposure. Second, as noted above, owing to a misstep in sample processing, the RBC PUFAs (especially) were variably damaged and had to be reconstructed based on experimental degradation studies and multiple imputation techniques as described in Pottala et al. [14]. Third, as discussed above, there was likely some misclassification due to the use of self-report for diabetic status. All of these factors added variability to the assessment of both exposures and outcome, which may alter the observed associations in various directions. Finally, these results apply to elderly (mean age 70), postmenopausal women; however, the robust findings associated with D5D and D6D ratios in racially and gender mixed populations suggests these results are generalizable to the general population (Table 1). The principal strengths of the study were its size, duration of follow-up, a well-characterized national cohort of women, an objective biomarker of fatty acid status, the evaluation of the full set of RBC fatty acids for relations with incident T2DM, and the inclusion of a sensitivity analysis to address potential reverse causation.

In conclusion, lower levels of RBC palmitic acid and the D6D ratio and higher levels of the D5D ratio were significantly and independently associated with incident T2DM over 11 years median follow-up in the WHIMS cohort. Whether there is a causal link between these fatty acid distributions and incident disease cannot be discerned from this study, but RBC fatty acid data may have value in stratifying patients for risk of T2DM.

## Supporting Information

S1 Table Number of subjects at risk by years of follow-up for selected RBC Fatty acid metrics (DOCX)

S2 Table Fully adjusted association of RBC fatty acid biomarkers (Omega-3 Index, Linoleic acid, PUFA Factor) on the risk of incident diabetes mellitus over 11-years median follow up. (DOCX)

S1 Text IRB information. (DOCX)

## Abbreviations

AA: Arachidonic acid (n-6)
D5D: Delta-5 desaturase ratio
D6D: Delta-6 desaturase ratio
DGLA: Dihomo-γ-linolenic acid (n-6)
LA: Linoleic acid (n-6)
n-3 (or -6): Omega-3 (or -6)
Omega-3 Index: RBC EPA+DHA
PH: Proportional Hazards
PUFA: Polyunsaturated fatty acid
RBC: Red blood cell
