# Supplementary material for: Red Blood Cell Fatty Acids and Incident Diabetes Mellitus in the Women’s Health Initiative Memory Study
Source: PLoS One. 2016 Feb 16;11(2):e0147894. doi: 10.1371/journal.pone.0147894 (PMC4755935; doi:10.1371/journal.pone.0147894)
Supplement: S1 Table — (DOCX) [file pone.0147894.s001.docx]

Supporting Materials

S1 Table. Number of subjects at risk by years of follow-up for selected RBC Fatty acid metrics

|  | Delta-6 Desaturase Ratio | | | | | | |
| --- | --- | --- | --- | --- | --- | --- | --- |
| Quintile | Year 0 | 2 | 4 | 6 | 8 | 10 | 12 |
| Q1 | 1275 | 1245 | 1201 | 1136 | 929 | 839 | 125 |
| Q2 | 1276 | 1251 | 1200 | 1130 | 957 | 883 | 138 |
| Q3 | 1276 | 1241 | 1192 | 1125 | 943 | 829 | 129 |
| Q4 | 1276 | 1230 | 1172 | 1103 | 918 | 820 | 125 |
| Q5 | 1276 | 1226 | 1154 | 1084 | 902 | 793 | 151 |
|  | Delta-5 Desaturase Ratio | | | | | | |
| Q1 | 1275 | 1223 | 1151 | 1077 | 893 | 791 | 123 |
| Q2 | 1276 | 1237 | 1183 | 1113 | 926 | 824 | 142 |
| Q3 | 1276 | 1245 | 1189 | 1118 | 934 | 847 | 123 |
| Q4 | 1276 | 1243 | 1196 | 1137 | 962 | 866 | 158 |
| Q5 | 1276 | 1245 | 1200 | 1133 | 934 | 836 | 122 |
|  | Palmitic Acid | | | | | | |
| Q1 | 1275 | 1244 | 1216 | 1164 | 970 | 881 | 150 |
| Q2 | 1276 | 1243 | 1194 | 1131 | 942 | 850 | 126 |
| Q3 | 1276 | 1240 | 1186 | 1126 | 946 | 850 | 142 |
| Q4 | 1276 | 1242 | 1174 | 1092 | 910 | 802 | 131 |
| Q5 | 1276 | 1224 | 1149 | 1065 | 881 | 781 | 119 |
