# Supplementary material for: Red Blood Cell Fatty Acids and Incident Diabetes Mellitus in the Women’s Health Initiative Memory Study
Source: PLoS One. 2016 Feb 16;11(2):e0147894. doi: 10.1371/journal.pone.0147894 (PMC4755935; doi:10.1371/journal.pone.0147894)
Supplement: S2 Table — (DOCX) [file pone.0147894.s002.docx]

S2 Table. Fully adjusted association of RBC fatty acid biomarkers (Omega-3 Index, Linoleic acid, PUFA Factor) on the risk of incident diabetes mellitus over 11-years median follow up

|  | Hazard Ratio | 95% Confidence Limits | P-value |
| --- | --- | --- | --- |
| Log(Omega-3 Index) per 1 SD | 0.98 | 0.90 – 1.06 | 0.59 |
| Log(Linoleic Acid) per 1 SD | 0.98 | 0.90 – 1.06 | 0.60 |
| PUFA Factor per 1 SD | 1.00 | 0.91 – 1.10 | 0.98 |

All models included 4 strata for hormone therapy trial randomization arm. The fully adjusted model included:

age, race, waist circumference, highest education, current smoking status, physical activity, weekly alcohol

intake, glycemic load, and family history of diabetes with categories listed in Table 2. All 10 imputed fatty

acid values were used for multiple imputation inference.
