# Supplementary material for: Red Blood Cell Fatty Acids and Incident Diabetes Mellitus in the Women’s Health Initiative Memory Study
Source: PLoS One. 2016 Feb 16;11(2):e0147894. doi: 10.1371/journal.pone.0147894 (PMC4755935; doi:10.1371/journal.pone.0147894)
Supplement: S1 Text — (DOCX) [file pone.0147894.s003.docx]

S3 Text

The Wake Forest University Institutional Review Board was the IRB of Record for WHIMS.
